# Bone Fragility Fractures in CKD Patients

**DOI:** 10.1007/s00223-020-00779-z

**Published:** 2020-11-21

**Authors:** Ana Pimentel, Pablo Ureña-Torres, Jordi Bover, Jose Luis Fernandez-Martín, Martine Cohen-Solal

**Affiliations:** 1 AURA Paris-Nord, Saint-Ouen, France; 2 Necker Hospital, University of Paris Descartes, Department of Renal Physiology, Paris, France; 3 Fundació Puigvert, Universitat Autònoma, IIB Sant Pau, REDinREN, Nephrology Department, Barcelona, Catalonia Spain; 4 Instituto de Investigación Sanitaria del Principado de Asturias (ISPA), REDinREN del ISCIII, Hospital Universitario Central de Asturias. Universidad de Oviedo, Bone and Mineral Research Unit, Oviedo, Asturias Spain; 5 INSERM U1132 & Université de Paris, Hôpital Lariboisière, Department of Rheumatology, Paris, France

**Keywords:** Bone, Fracture, Bone mineral density, CKD-MBD, Phosphate, Calcium, Parathyroid hormone, Imaging

## Abstract

Chronic kidney diseases (CKD) are associated with mineral and bone diseases (MBD), including pain, bone loss, and fractures. Bone fragility related to CKD includes the risk factors observed in osteoporosis in addition to those related to CKD, resulting in a higher risk of mortality related to fractures. Unawareness of such complications led to a poor management of fractures and a lack of preventive approaches. The current guidelines of the Kidney Disease Improving Global Outcomes (KDIGO) recommend the assessment of bone mineral density if results will impact treatment decision. In addition to bone density, circulating biomarkers of mineral, serum bone turnover markers, and imaging techniques are currently available to evaluate the fracture risk. The purpose of this review is to provide an overview of the epidemiology and pathogenesis of CKD-associated bone loss. The contribution of the current tools and other techniques in development are discussed. We here propose a current view of how to better predict bone fragility and the therapeutic options in CKD.

## Introduction

The high morbidity and mortality rates observed in progressive chronic kidney disease (CKD) are tightly associated to the underlying metabolic bone alterations. The mineral and bone disorders (MBD) associated with CKD include bone, biochemical, and cardiovascular abnormalities in the same entity since they share common pathophysiological mechanisms. This current new definition of CKD-MBD aims at a better awareness of concommitant bone and cardiovascular events and shows that common molecules are involved in both tissues breakdown. The initial characterization of previously called renal osteodystrophy (ROD) was based on bone biopsy and is extended nowadays to bone markers of fragility, including biochemistry and imaging. Bone fragility includes all the aspects that lead to fractures, particularly bone volume, structure, rate of remodeling, as well as mineralization defects which can be also observed in CKD. Fractures are the end results of skeletal fragility, the prevention of which being a major objective. The evaluation of fracture risk is required in light of high mortality risk and hospitalization costs related to fracture in dialysis patients [1–5], which enhances the economic burden of CKD-MBD.

An important contribution of the Kidney Disease Improving Global Outcomes (KDIGO) 2017 revised version [6] compared to 2009 KDIGO CKD-MBD guidelines [7] is to recommend the assessment of bone mineral density (BMD) in case a treatment is considered, thus highlighting the need of a better assessment of CKD patients suffering from skeletal fractures. However, there is no recommendation about which BMD should be assessed and at which frequency in CKD patients as it is in osteoporotic postmenopausal women. Indeed, in this population, a second BMD performed 3 years after the initial measurement was not associated with improved discrimination between women who did and did not experience subsequent hip fracture or major osteoporotic fracture beyond the baseline BMD value alone and should not routinely be performed [8]

## Epidemiology of Skeletal Fractures in CKD

The life expectancy of CKD patients is constantly improving because of a better prevention and management of complications. The prevalence of skeletal fractures in the general population as well as in subjects with CKD has significantly increased in the recent years along with aging and the related increase in bone loss. This can be evidenced by several longitudinal studies reporting the incidence of fractures during the last decade [3, 9–27]. They found the fracture incidence to raise progressively from 15.0 to 20.5, 24.2, 31.2, and 46.3/1000 person-years for CKD stages 1 to 2, 3a, 3b, and 4, respectively [28]. The risk of skeletal fracture is up to 5 times higher in individuals with an estimated glomerular filtration rate (eGFR) < 15 versus > 60 ml/min/1.73 m^2^. This is particularly true in CKD patients older than 65 years who show the highest rate of fractures, with 1/10 women and 1/20 men experiencing at least one fracture in the subsequent 3 years of follow-up [29]. In a recent Dialysis Outcomes and Practice Patterns Study (DOPPS) report, the incidence of skeletal fractures was significantly higher for patients receiving hemodialysis therapy than in the general population with a 3.7-fold increase of the unadjusted relative risk of death [4].

Peripheral fractures are the best documented. The incidence of hip fractures is four-fold higher for dialysis patients than for the general population after adjustment for age, gender, and ethnicity [9, 30]. Moreover, the incidence of hip fracture in CKD patients differs by ethnicity and sex, being 3 times higher for Caucasian than African-American [1] and twice higher for women than men [3, 21, 27, 31]. They are also associated with several risk factors including older age, low body mass index, and long dialysis vintage, as well as with a history of prior hip fracture [23]. A recent study evaluated the association between CKD stages G3–G5 versus eGFR > 60 ml/min/1.73 m^2^ and the risk of a new non-hip fracture or fragility fracture in patients with a first hip fracture. It found that the risk of a subsequent major non-hip fragility fractures following hip fracture was not increased in patients with CKD G3–G5 compared to eGFR > 60 ml/min/1.73 m^2^. Mortality risk was higher in both hip fracture and non-hip fracture patients with CKD G4 and G5 [32].

The most recent and the largest epidemiologic study assessing the incidence and the risk factors associated with hip fractures is a South Korean report from 352,624 CKD adult subjects [33]. They found that lower eGFR and high urine albumin levels were associated with a higher risk of hip fracture. The HR for hip fracture was 1.89 among participants with eGFRs of 30–44 and 15–29 mL/min/1.73 m^2^ relative to those with an eGFR ≥ 60 mL/min/1.73 m^2^, respectively. The HRs were 1.30 for moderate albuminuria and 1.58 for severe albuminuria. Participants with albuminuria had a higher risk of hip fracture than those without albuminuria, even when they belonged to the same eGFR category. The effects of each 10 mL/min/1.73 m^2^ decrease in eGFR were stronger with advancing albuminuria severity.

The US Medicare data for hemodialysis patients have recently reported a downward hip fracture incidence trends from 2000 to late 2009 [1], most prominent in older adults of both genders [34]. Indeed, the incidence of hip fracture increased when dialysis treatment was initiated from 1996 to 2004 and then declined until 2009, although it remained higher than in 1996 [16]. The relative risk of hip fractures starts to increase as early as age 55 years and is more increased for CKD patients with high bone turnover disease rather than with low bone turnover disease [23, 27]. The risk of skeletal fractures combines classical risk factors that are applied for osteoporosis in addition to those associated with CKD. After 4 years of dialysis, the age-standardized incidence ratio of hip fracture was 9.83 (8.61, 11.2; 95% C.I.) for men and 8.10 (7.23, 9.07; 95% C.I.) for women [9]. Even in renal transplant recipients, prior dialysis vintage is associated with increased risk of hip fracture [2, 35]. Because of a rare collection of vertebral fractures, the prevalence of this major bone fragility is poorly documented in cohort and dialysis registers. This has been reported to be low, ranging from 7 to 20% [36, 37], and only 1% of patients appear to have vertebral fractures based on clinical diagnosis in a single clinical trial [38]. It is likely that this is underestimated because of the lack of systematic radiographic imaging or in the presence of spinal pain or the difficulty to differentiate a mild vertebral fracture from deformities as seen in Scheuermann disease. A more recent analysis of a prospective cohort of 612 patients with 3‒5 stage CKD revealed 18% of vertebral fractures. After a 3-year follow-up, the prevalence of fractures was correlated with a poor survival and an independent predictor of all-cause mortality [39].

The risk of mortality following a hip fracture is significantly high in CKD patients, [40, 41] but has essentially remained unchanged since 1998 for either sex [31]. The thirty-day mortality rate after hip fracture is of 16% for CKD patients initiating dialysis and age > 67 years in the United States [16]. The hip fracture-related mortality risk is also two times higher with eGFR < 45 than ≥ 45 ml/min/1.73 m^2^ [42]. In the French national database in 2010, a significant higher mortality rate was observed after hip fracture in patients on dialysis therapy compared to those without dialysis, up to 12% for men and 8% for women, as well as a longer hospital stay in the intensive care unit [3]. Several major risk factors were found to be associated with those hip fractures, particularly the presence of cardiovascular diseases and dementia.

## Evaluation of Fracture Risk in CKD-MBD

Bone strength is based on both the quantity and quality of bone, influenced by several factors such as the level of bone remodeling or the matrix composition. Bone quantity can be estimated by bone density while bone quality by the microarchitecture, both leading to a reduced mechanical bone adaptation. Thus, most fractures occurring after a fall or a low kinetics trauma take place in a bone with low mechanical strength. Cumulative studies revealed that the high fracture risk results from the combination of CKD-induced changes in bone and mineral metabolism in addition to the classical fracture risk factors observed in the non-CKD population including age, gender, diabetes, and glucocorticoid use. Several imaging and biochemical markers, here below described, have demonstrated their usefulness to assess the fracture risk in CKD population.

### Bone Mineral Density

In non-CKD patients, the reduction of BMD as measured by dual-energy x-ray absorptiometry (DEXA) predicts the fracture risk. Osteoporosis is defined by a T-score below 2.5 standard deviations (number of standard deviations of BMD below the mean BMD for the young healthy population). The relevance is limited in patients with scoliosis, osteoarthritis at the lumbar spine, and noteworthy in the presence of vascular calcifications that are frequent in CKD and which overestimate the BMD. Another limitation of BMD and T-score is that it does cannot distinguish the type of ROD and cannot discriminate osteoporosis as a low bone mass than low bone density related to mineralization defect. A meta-analysis showed that BMD is lower in pre-dialysis and dialysis CKD patients with fractures compared to those without [43]. Despite these findings, BMD measurement was not recommended in the 2009 KDIGO guidelines because of inconsistency in the results obtained from most of cross-sectional studies and the mild reduction of BMD that cannot be used at the individual level. More recently, prospective cohort studies showed good predictive value of BMD for risk of peripheral fracture and hip fractures in CKD stages 3-5D [12, 28, 44, 45]. This prompted the recent 2017 KDIGO guidelines to recommend BMD measurement if results will impact treatment decisions, as advocated by the World Health Organization (WHO) for the general population.

Nevertheless, scarce data are available for the prediction of vertebral fractures, one of the main hallmarks of bone fragility. Case–control studies with a small number of dialysis patients revealed that a low BMD at the lumbar spine was associated with vertebral fractures and with the prevalent or self-reported peripheral fractures [46]. In a meta-analysis of 13 studies [43], only one mentioned a relationship between vertebral fracture and the dialysis vintage, but did not find any association between BMD and the risk of fracture [11]. Albeit a lower discriminative value of BMD in CKD, recommendations of BMD measurement for predicting peripheral fractures draw the attention to a better assessment of a well-recognized marker of fragility fractures. In addition, the Fracture Risk Assessment Tool (FRAX) index is sufficient for 10-year prediction of major osteoporotic fractures, assessing clinical risk factors together with BMD [44, 47], in particular in older patients with CKD stages 2–5 [47]. A recent study in 718 Polish hemodialysis patients, who were followed up for two years, showed that the Polish version of FRAX > 5% (without the DXA examination) and some particular variables of the FRAX calculator had a sensitivity of 70.0% and a specificity of 69.8% as the prognostic threshold for major bone fractures. Again, in this study, FRAX sensitivity for bone fracture prediction was significantly higher, but specificity is lower than those of FRAX ≥ 10%, used in general Polish population. The reason for this can be an underestimation of bone fracture risk with FRAX in dialysis patients [48].

Bone diseases related to CKD represent a large spectrum of histological features, all associated with a high risk of fracture. Indeed, low BMD may be observed in osteoporosis, but also in hyperparathyroidism, osteomalacia, and adynamic bone diseases. This difficulty of characterizing the bone disease requires additional evaluation in order to address specific treatment of the cause.

### Macro and Microarchitecture of Bone

Altered hip geometry, as derived from DEXA hip measurement, has been associated with the risk of fracture. Non-CKD women with hip fractures have thinner femoral cortices and longer femoral-neck axis length than women without fractures. Such DXA-derived cortical parameters were reported in patients in CKD patients [49]. Although hip structural values are correlated with BMD, whether they provide additional information independent of BMD and if this improves fracture prediction remains unknown [50]. Further investigations using the new three-dimensional (3D)-DXA software will be useful in CKD as previously shown in patients with primary hyperparathyroidism [51].

Another marker emerged recently to evaluate bone strength. The *Trabecular Bone Score (TBS)* is a gray-level textural index derived by an algorithm that analyzes the spatial organization of pixel intensity from lumbar spine DEXA images. TBS is not a direct measurement of bone microarchitecture, but it is correlated to it. TBS can be used to predict fractures independent of major clinical risk factors or a real BMD measured in the general population [52]. In a cohort of 1426 participants older than 40 years and followed for a mean of 4.7 years, including 199 patients with eGFR < 60 ml/mn/1.73 m^2^ (72.4% CKD stage 3a, 25.1% CKD stage 3b, and 2.5% CKD stage 4), low lumbar spine TBS was independently associated with increased fracture risk when kidney function was low, providing a new tool to better identify the patients at risk [53].

As discussed above, BMD measurement by DEXA is insufficient to assess the fracture risk partly because of a weak discriminating power between cortical and trabecular bone. Indeed, bone strength depends on cortical bone which is highly altered in CKD. Bone biopsies obtained from CKD patients revealed that low bone turnover is associated with normal cortical porosity, while high serum PTH levels are present with normal cortical thickness and high trabecular bone volume [54]. However, the biopsies performed were not systematic, but for research purposes, introducing a recruitment bias for a clear-cut interpretation. Nevertheless, assessment of cortical bone structure in CKD is crucial since predominantly affected by bone loss and highly associated with peripheral fractures at any skeletal sites [55]. Undoubtedly, such structural components are additional parameters to be included in the treatment decision in addition to the rate of bone remodeling and bone and mineral biomarkers.

Quantitative computed tomography (QCT) revealed a higher number of patients experiencing bone loss at the hip as compared with BMD measured by DEXA [56]. The peripheral QCT device from Stratec, which measures only BMD at the mid-radius and then mainly cortical bone, showed that high PTH, long dialysis vintage, and cortical BMD were significant predictors of skeletal changes [57]. BMD and microarchitecture assessed by high-resolution peripheral quantitative tomography (HRpQCT) allow a separate measurement of cortical and trabecular bone [58] and help to determine the underlying mechanisms of bone loss [59]. In CKD stages 2–4, HRpQCT showed early impairment of trabecular bone, before the onset of secondary hyperparathyroidism (SHPT). This could explain in part the high risk of fractures in early CKD [60] but also in patients with long CKD history [58]. HRpQCT measurements allow to distinguish between cortical and trabecular bone density in vivo. It assesses both bone volume and density in the same compartment, thus potentially yielding information regarding bone mineralization as well. A recent study in 68 dialysis children with bone biopsy determination showed that 76% of patients had normal/high bone turnover, 13% had adynamic bone disease, and 11% had osteomalacia. Bone formation rate did not correlate with any HRpQCT determinations. Bone volume measurements were highly correlated between bone histomorphometry and HRpQCT (bone volume/tissue volume between the two techniques) [61]. We have also recently analyzed bone trans-iliac biopsies performed for vertebral or hip fracture in 12 adult dialysis patients and compared with bone microarchitecture assessed by two-dimension histology (2D) and in vitro microcomputed tomography (3D-µCT) as well as with microarchitecture data obtained from HRpQCT. We found that 3D-µCT was a reliable method for the measurement of cortical bone in bone biopsies. However, only HRpQCT allows discriminating patients with multiple fractures [62].

Bone loss observed in CKD stage 5D affected cortical BMD and thickness which is correlated with high PTH and dialysis vintage, but not with trabecular bone [55]. However, levels of neither calciotropic hormones such as PTH nor bone remodeling markers were associated with changes in trabecular density, number, and heterogeneity. These data have been challenged more recently by a study using bone biopsy data that correlated cortical BMD to biochemical markers [63]. Cortical BMD was negatively correlated to serum PTH, TRAP5b (tartrate-resistant acid phosphatase 5b), and BSAP (bone-specific alkaline phosphatase) levels, suggesting that low remodeling rate is associated with higher bone density and strength. These controversial data are likely related to changes that occur at different remodeling rates, biomarkers reflecting the level of bone remodeling at short term while cortical thickness integrating remodeling rates for a long period. Research should address new imaging of the appendicular skeleton to estimate cortical bone loss [58]. For example, analysis of bone matrix, including collagen and mineral properties, should be developed [64, 65]. Despite the great interest of predicting the risk of fracture and identifying people who will benefit from therapeutic interventions, most of these new tools are not widely available, as well as evidence of additional interest compared to DEXA [66].

#### Bone Biomarkers

Since several decades, PTH remains the best surrogate biomarker to evaluate the level of bone remodeling in CKD. Most CKD patients with adynamic bone disease displayed serum PTH level < 150 pg/mL [67] and those with histological SHPT show PTH values > 600 pg/ml [68]. Although within the KDIGO recommended PTH target of 2–9 the normal range, those values within the target range do not discriminate the different forms of ROD [69] and both high and low circulating PTH levels can be associated with high fracture rate and mortality risk [4, 5, 20, 23, 36, 70–72]. Interestingly, serum PTH levels, just before the occurrence of a new fracture, are associated with the increased risk of fracture, in contrast to baseline or time-averaged serum PTH levels. The upper and lower PTH values of the U-shaped PTH curve are associated with a significantly increased risk of fracture as compared with PTH values within the recommended NKF/K-DOQI target values [12]. Therefore, treatment should not be based on a single PTH value but rather on a trend of PTH within the previous months. As mentioned in the KDIGO 2017 guidelines, “persistently” and “progressively rising” PTH level should be considered, rather than “above the upper normal limit”. Total or subtotal parathyroidectomy reduces serum PTH levels and subsequently bone turnover, improves BMD and reduces long-term risk for fractures in CKD stage 5D patients [73–75]. Other circulating bone biomarkers provide additional information of the rate of bone turnover and microstructure of bone. These include cross-linked collagen type I peptide (CTX) and tartrate-resistant acid phosphatase 5B (TRAP5b for bone resorption, as well as bone-specific alkaline phosphatase, and procollagen type 1 N-terminal pro-peptide (P1NP) for bone formation [56]. A recent study has shown the predictive value of association of these markers and PTH to predict bone fragility [76]. In another recent study, not yet investigated for fracture prediction, a panel of 4 microRNAs was superior to circulating concentrations of bone-specific alkaline phosphatase and CTX for discriminating low bone turnover within individual cancellous, endocortical, and intracortical bone compartments [77].

The phosphate/FGF23/alpha-Klotho axis is another determinant of bone mineralization in CKD. Circulating phosphate levels slowly increase with the progression of CKD and both directly and indirectly contribute to the skeletal fragility associated with CKD-MBD, in part via the stimulation of PTH and FGF23 production [78]. The role of serum phosphate as a bone fracture risk factor has been poorly studied. In general population, the results from the Dutch Rotterdam Study (RS-I, RS-II and RS-III) and the US Osteoporotic Fractures in Men (MrOS) study including 12,216 patients followed up to 10.9 years showed that serum phosphate was significantly associated with bone fractures [79]. Quintiles 4 (3.3–3.5 mg/dL) and 5 (> 3.5 mg/dL) of serum phosphate were associated with a higher risk of incident of all types of fractures compared with quintile 1. In this study, serum phosphate was associated with fracture risk in subjects without CKD and also in men with CKD. In men with CKD the association between serum phosphate and fracture risk was even stronger than in non-CKD subjects. In 2004, Block el al. found that serum phosphate was significantly associated with bone fracture-related hospitalization with a relative risk of 1.12 per mg/dL increase in serum phosphate in hemodialysis patients [80]. Unpublished data from the COSMOS study (Current management Of Secondary hyperparathyroidism: a Multicentre Observational Study) are in the same line, showing, in a period of 3-year follow-up, that high serum phosphate is associated with a higher incidence of bone fractures. By contrast, Aleksova et al [81] found that low serum phosphate was associated with a higher risk of bone fractures in transplanted patients.

Serum FGF23 levels significantly increase in early CKD stages and coincide with the decrease of 1,25OH2D [82]. FGF23 is mainly produced by osteocytes and osteoblasts and exerts its major physiological actions in the kidney, stimulating urinary phosphate excretion and inhibiting calcitriol synthesis after binding to a complex formed by alpha-klotho and canonical FGF receptors. FGF23 plays an important role in regulating bone mineralization. In CKD stage 5D patients, BMD is not correlated with serum FGF23 levels [83]. However, high FGF23 and low klotho levels were found in CKD patients with type 2 diabetes compared to controls and even further in CKD with fractures [84]. High FGF23 was associated with reduced osteoid thickness in children both with normal renal function and dialysis [85], through the regulation of tissue non-specific alkaline phosphatase activity (TNAP) via FGFR-3, vitamin D, and klotho-independent manner. Finally, excessive FGF23 contributes to bone loss in CKD via an alpha-klotho-dependent mechanism and the stimulation of the osteoblast Wnt inhibitor Dkk1 [86]. Therefore, inactivation of the Wnt/b-catenin signaling pathway by the altered phosphate/FGF23/Klotho axis may provide another autocrine/paracrine mechanism favoring bone loss in CKD-MBD.

### Contribution of Bone Histology

Bone histomorphometry is the gold standard to evaluate bone abnormalities of CKD-MBD [87], although not routinely recommended because of its invasive nature and the limited number of specialized laboratories. Histological signs of high bone turnover are expected in that 85–90% of patients have increased serum PTH level in CKD stages 3–5 [88, 89]. Accordingly, histological high bone turnover in 47.2% of patients with CKD stages 3–4 and in 61.4% with CKD stage 5 [90]. However, low-turnover bone disease has also been reported as being the predominant pattern in 2 small populations with pre-dialysis and a wide eGFR range (< 5 to 90 ml/min/1.73 m^2^) [91, 92] and in most CKD patients on dialysis [54, 93]. These conflicting results might be related to the recruitment of patients; bone biopsies being mostly performed in symptomatic patients with no systematic analysis for epidemiologic purposes are influenced by several confounding factors. Moreover, the lack of clear-cut and quantitative definition of adynamic bone disease (ABD) and the absence of previous labeling do not allow drawing clear conclusions.

In addition, CKD progression might result in changes from low bone turnover at earlier stages to high bone turnover features at later stages [94] and there is no association between fracture incidence and the histological type of bone diseases. Finally, bone biopsy may show mineralization defects and osteomalacia, even in the absence of marked changes in circulating biomarkers. Actually, the first causes of bone mineralization defect are vitamin D deficient-related osteomalacia, hypophosphatemia, and aluminum overload. Osteomalacia remains present in long-term dialysis therapy and remains a cause of fractures. Moreover, the use of the new bone nomenclature (TMV), which now adds bone mineralization indices to turnover and volume should contribute to a better definition of the classical renal osteodystrophy and a better estimation of fracture risk and understanding clinical features.

## Therapeutical Management of Fractures in CKD

Several medications have been shown to reduce the fracture rate in patients with low BMD. All of them can be proposed for the prevention of fractures when eGFR is above 30 ml/min/1.73 m^2^ after correction of 25OHD insufficiency. In any case, supplementation of vitamin D is strongly recommended when initiate therapies regardless of kidney function. Specific cause of non-osteoporotic fractures should be discarded and required specific treatments. The presence of osteomalacia can be suggested by low serum 25OHD levels, long-lasting hypocalcemia or hypophosphate, and high alkaline phosphatase levels [95]. All the medications that received the approval for the prevention of fractures will follow the same recommendations as in non-CKD patients. This is the case of raloxifene, bisphosphonates, and denosumab, the three of them being pure inhibitors of bone resorption while teriparatide, the human recombinant PTH, increased bone resorption and formation with a positive bone balance in favor of bone formation. Treatment of CKD stage 4-5D patients with osteoporosis requires more caution and should be discussed after the correction of mineral biomarkers. There is no evidence that any treatment prevents the fracture rate in patients with low BMD alone. Nevertheless, the presence of major osteoporotic fracture occurring in skeletal sites such as vertebrae, pelvis, humerus and hip demonstrates a bone fragility that requires a more active treatment.

Most anti-fracture treatments are contra-indicated in patients with an eGFR < 30 ml/min. Bisphosphonates accumulate in bone tissues, this being promoted with reduced renal clearance and subsequently might induce defects of bone mineralization and osteomalacia [96]. Albeit there is no evidence of such a deleterious effect, the use of bisphosphonates requires being aware of such possible complication. Few studies and early post hoc analyses showed increased BMD with bisphosphonates, but a systematic review suggested that no evidence that these treatments could be helpful in CKD as no anti-fracture effect has been shown [97]. Denosumab, an anti-RANKL biologic therapy not cleared by the kidney, offered new opportunities for managing fractures. In a subgroup of 73 women with CKD stage 4, denosumab increased the BMD in a similar manner than in women with CKD stages 1–3, but the low number of patients did not allow to demonstrate an effect in fracture prevention [98]. In patients with CKD stages 4-5D with severe SHPT, denosumab promoted marked hypocalcemia and increased PTH level within the first 15 days of its administration, which often required calcium supplementation. Therefore, a close monitoring is recommended during those first weeks in patients with advanced CKD. A trial compared the one-year effect of denosumab to alendronate in dialysis patients with low PTH and found that there was a better increase in BMD and improvement of bone biomarkers associated with denosumab [99]. In the absence of linear relation between BMD and fracture risk in CDK, the increase in BMD does not fully secure the effect on the prevention of the risk of fracture at short and long term. Further trials are needed to test the effect of such a medication in both BMD and fracture risk.

**Fig. 1:**
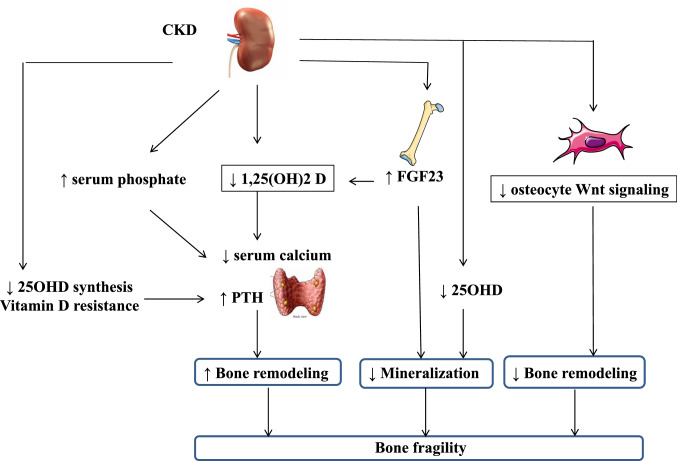
Pathophysiology of fractures in CKD. Schematic representation of the changes induced by CKD: 25OHD 25hydroxy-vitamin D; 1,25(OH)2 D: 1–25 dihydroxy-vitamin D. PTH parathyroid hormone; Wnt wintless

**Fig. 2:**
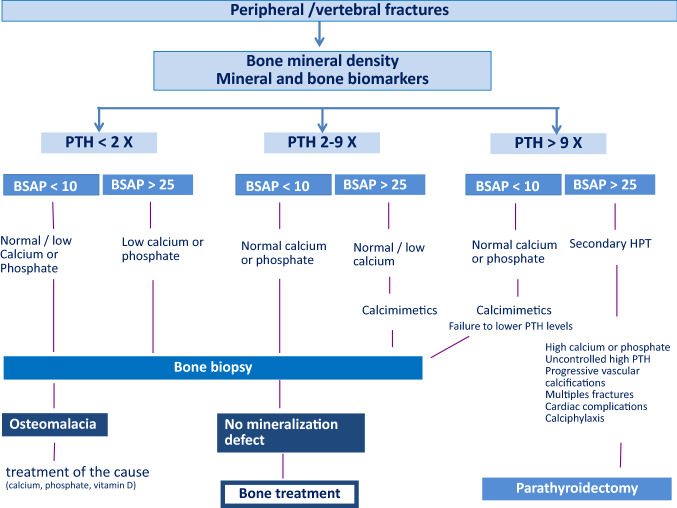
Guidelines for the management of fractures. A fragility fracture requires the measurement of bone mineral density and a deep analysis of mineral metabolism markers. The first step aims to rule out an osteomalacia with the use of bone biomarkers and if possible a bone biopsy. Guidelines are proposed as a function of PTH levels. Reduction of PTH should be achieved before the introduction of any anti-resorbing agents. BSAP bone-specific alkaline phosphatase

Recommendations for the management of fractures in CKD patients rely more on clinical experience than on scientifically well-proven evidence (Fig. 1). First characterization is based on the BMD measurements, followed by the PTH and bone-specific alkaline phosphatase levels. In patients with high PTH, the reduction of SHPT improved other biological parameters and the bone status. Indeed, accounting for differences in baseline characteristics, multiple fractures, and/or events prompting discontinuation, oral cinacalcet efficiently reduced the rates of clinical fractures in elderly dialysis patients [38]. In these patients, denosumab might be discussed once PTH target has been achieved and that BMD remains significantly low [100]. The indication of a subtotal surgical parathyroidectomy should also be discussed in case of persistent and uncontrolled hypercalcemia, hyperphosphatemia, and high serum PTH levels, as well as in cases of progressive vascular calcifications, calciphylaxis, multiple skeletal fractures, and cardiac complications (Fig. 2).

The more challenging situation is the treatment of patients with low PTH. The restoration of PTH levels to the “2–9 time-fold” target is recommended to ensure a proper function of bone cells and a replacement of old to new bone matrix. This could be achieved by reducing calcium load either through the diet or lowering calcium content in the dialysate [101]. More specific anti-resorbing therapy may promote adynamic bone disease and the emergence of vascular calcifications. However, denosumab does not induce any aortic calcifications in postmenopausal women without renal failure [102] and there are no data in humans showing that reducing bone turnover would increase the risk of vascular calcifications. In addition, denosumab reduces the deposition of calcium in vessels of osteopenic mice [103]. Further studies are needed to determine whether this drug has an impact on vascular calcifications in CKD.

To the best of knowledge, the optimal therapy for CKD-associated osteoporosis would be a drug that enhances bone formation and restores bone mass. In this sense, the effect of teriparatide has been evaluated in a small pilot study including 7 hemodialysis patients. There was a significant increase in lumbar and femoral BMD after 6 months of treatment and in 6 out of the 7 patients [104]. However, we are lacking evidence regarding the effect of teriparatide on the prevention of fractures in advanced CKD. Anti-sclerostin antibody is a promising anabolic agent as it promotes bone formation by binding to sclerostin, a natural antagonist of Wnt signaling. Indeed, romosozumab increased BMD and prevents bone fractures in postmenopausal women without CKD [105]. Interestingly, anti-sclerostin antibody increases bone formation and bone mass in rats with CDK only with low, but not with high PTH [106]. Trials based on this promising therapy in CKD are highly awaited taking into account the alert on the possible cardiovascular side effects.

In conclusion, the high incidence of fractures and mortality in patients with CKD requires a better evaluation of fracture risk, as well as more randomized controlled trials on fracture prevention. Because of the multiple aspects of bone fragility in CKD, a multidisciplinary discussion including renal and bone experts in CKD-MBD would be desirable before any initiation of specific bone treatments.
